# Surface interactions of gelatin-sourced carbon quantum dots with a model globular protein: insights into carbon-based nanomaterials and biological systems†

**DOI:** 10.1039/d4na00842a

**Published:** 2024-12-19

**Authors:** Shima Masoudi Asil, Mahesh Narayan

**Affiliations:** a The Department of Environmental Science & Engineering, The University of Texas at El Paso El Paso TX 79968 USA; b The Department of Chemistry & Biochemistry, The University of Texas at El Paso 500 W. University Ave. El Paso TX 79968 USA mnarayan@utep.edu

## Abstract

Carbon nanomaterials (CNMs), such as carbon nanotubes (CNTs), graphene quantum dots (GQDs), and carbon quantum dots (CQDs), are prevalent in biological systems and have been widely utilized in applications like environmental sensing and biomedical fields. While their presence in human matrices is projected to increase, the interfacial interactions between carbon-based nanoscopic platforms and biomolecular systems continue to remain underexplored. In this study, we investigated the effect of gelatin-sourced CQDs on the globular milk protein beta-lactoglobulin (BLG). Exposure to the CQDs resulted in the disruption of BLG's tertiary and secondary structural elements (transformation of isolated helices to coiled-coils and increased beta-sheet content), with IR amide backbone signatures further confirming CQD-induced alterations in protein structures. Importantly, the structural perturbations induced by CQDs compromised BLG : retinol interactions, potentially affecting its physiological ligand transport function. By contrast, cytotoxicity analyses revealed a high viability of neuroblastoma cells exposed to this CNM, suggesting biomolecule-specific effects. Collectively, the data reveal aberrant molecular and functional consequences associated with the interactions of a globular protein with an otherwise biocompatible CQD. In conclusion, this work represents the initial steps toward a comprehensive understanding at the atomic and molecular levels of the outcomes linked to the utilization of carbon-based nanomaterials and their potential adverse systemic consequences.

## Abstract 2

This study explores interactions between gelatin-based carbon quantum dots (CQDs) and a model protein, shedding light on the behavior of carbon nanomaterials in biological systems and their biomedical potential.

## Graphical abstract

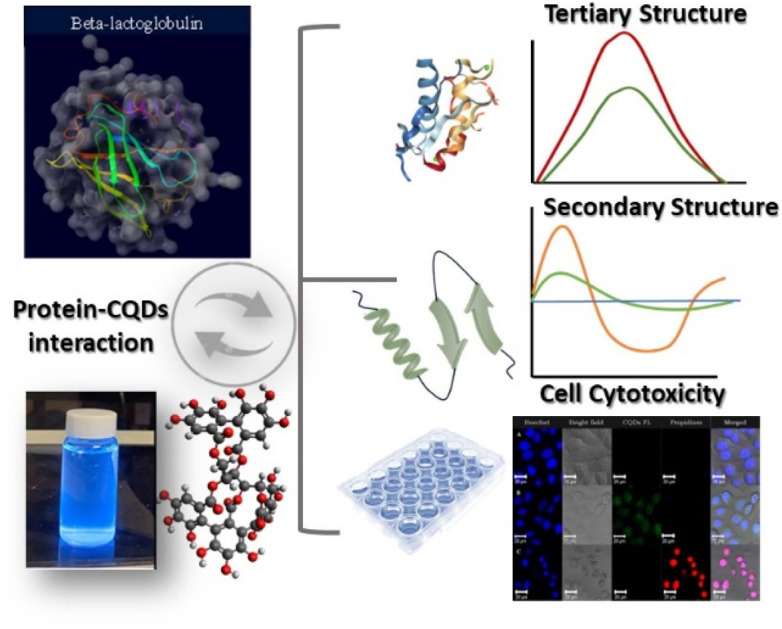

## 1 Introduction

The rapid evolution of nanotechnology raises the prospect of nanoparticles interacting with both humans and the environment.^1–3^ CNMs, CNTs, GQDs, carbon dots (CDs), CQDs, carbon nano-onions (CNOs), fullerenes, *etc.* have found applications in the environment (sensors), agriculture (nano-fertilizers), and biomedicine (gene therapy, drug-delivery).^4–7^ Some specifics of their application in the biological arena include the application of single-walled CNTs–siRNA conjugates that have been used to silence specific genes in human T-cells and primary cells. It is notable that these cell types were unresponsive to liposome-based nonviral vectors.^8^ CNT-reinforced coatings demonstrated potential in orthopedic applications involving knee and hip replacements.^9^ CDs have been extensively used for optical imaging *in vivo*.^10^ Modified CQDs, GQDs, and various other carbon nanomaterials exhibit the capability to penetrate the blood–brain barrier (BBB), rendering them highly appealing for prophylactic and therapeutic purposes.^11–14^ Furthermore, in mouse brains, GQDs mitigated dopamine α-synuclein fibril-induced neuronal loss and mitigated compromised behavioral outcomes.^15^ GQDs have also been found to ameliorate the aggregation of human islet amyloid precursor protein (IAPP) and reduce its toxicity to zebrafish.^16^ Recently, citric acid-derived CQDs prevented the soluble-to-toxic transformation of hen egg white lysozyme, mitigated paraquat-induced death in neuroblastoma-derived cell lines, and reduced neurotoxicant-related mortality rates in a *Caenorhabditis elegans in vivo* model.^17^

Their small size, biocompatibility, sensitivity, reactive oxygen species (ROS) scavenging ability, intrinsic fluorescence properties, capacity to traverse the blood–brain barrier, and amenability to chemical tuning make CNMs particularly attractive for the aforementioned applications.^18–20^ However, despite the extensive use of nanoparticles such as CNMs in biological scenarios, there is limited knowledge and understanding of their impact on cellular constituents, including proteins, nucleic acids, lipids, and carbohydrates. The tiny size and higher surface/volume ratio in these nanoparticles provide higher surface energy compared to bulk materials. These properties of nanoparticles facilitate their absorption into biomolecules such as RNA, DNA, lipids, and proteins upon their introduction into biofluids and, consequently, enhance the risk of disruption in their functionality and structure.^21^ This is important not only for advancing the clinical and pharmaceutical use of CNMs but also for designing 2nd and 3rd generation carbonaceous interventional platforms.^22^ There are many reports demonstrating that nanoparticles can act as an effective inhibitor against the fibrillation of various proteins.^23^ This protein fibrillation is associated with many neurodegenerative diseases like Parkinson's and Alzheimer's. However, limited studies have revealed the negative impacts of these nanomaterials on protein conformations. Nanoparticles (NPs) have been found to induce conformational changes in proteins, promoting fibril formation and oligomer formation on the NP surface, as demonstrated with various NPs such as copolymers, ceria, carbon nanotubes, and quantum dots.^24–29^ Nanocarbon materials like CNTs, graphene, and graphene oxide (GO) are widely explored for biomedical applications such as drug delivery and bioimaging. Understanding their interactions with proteins is critical, as these interactions can have both beneficial and adverse effects. For instance, GO has been shown to interact with human plasma proteins and regulate cellular processes, but it can also cause severe lung toxicity in animal models.^30–32^ Similarly, CNTs can bind to pulmonary surfactant proteins, increasing susceptibility to lung infections, while functionalized CNTs have been shown to inhibit enzymatic activity or enhance protein stability and enzymatic activity.^33^ These interactions depend on nanoparticle surface properties and preparation methods, highlighting the need for further research to ensure safe and effective applications in biomedicine.^34^ These findings highlight the potential impact of NPs on the disruption of protein structure and function.^35,36^ Additionally, the NP surface may induce thermodynamic instability to adsorbed proteins, leading to susceptibility to chemical denaturation; for instance, ZnO NPs induced unfolding of the signal-transduction ToxR protein and caused significant changes in protein conformations upon binding ZnO NPs.^27,37,38^ Understanding the behavior of proteins on NP surfaces requires further investigation due to their diverse chemical properties.^39–42^ Furthermore, it is also possible that CNMs might also interact with non-target cellular metabolites, necessitating a sound atomic and molecular-level understanding of their effect on the structure and functionality of the aforementioned classes of biomolecules.^43^

To address the impact of CNMs on protein structure and function, we examined the interfacial interactions between gelatin-derived CQDs and the small globular milk whey protein beta-lactoglobulin (BLG) using spectroscopic techniques and a binding affinity assay. It is noted that gelatin-derived CQDs have been found to be biocompatible, possess free radical scavenging ability, rescue cell lines from apoptosis and necrosis, be amenable to bio-imaging due to their high quantum yield, and easily be surface-functionalized for expanded applications.^17,36,44,45^ They can serve as effective nanocarriers for drugs and bioactive molecules due to their non-toxic biodegradability *in vivo*, as well as their significant potential for surface chemical alteration and cross-linking capabilities, thereby enhancing the targeting precision and therapeutic efficacy of chemotherapeutic agents.^46,47^

**Fig. 1:**
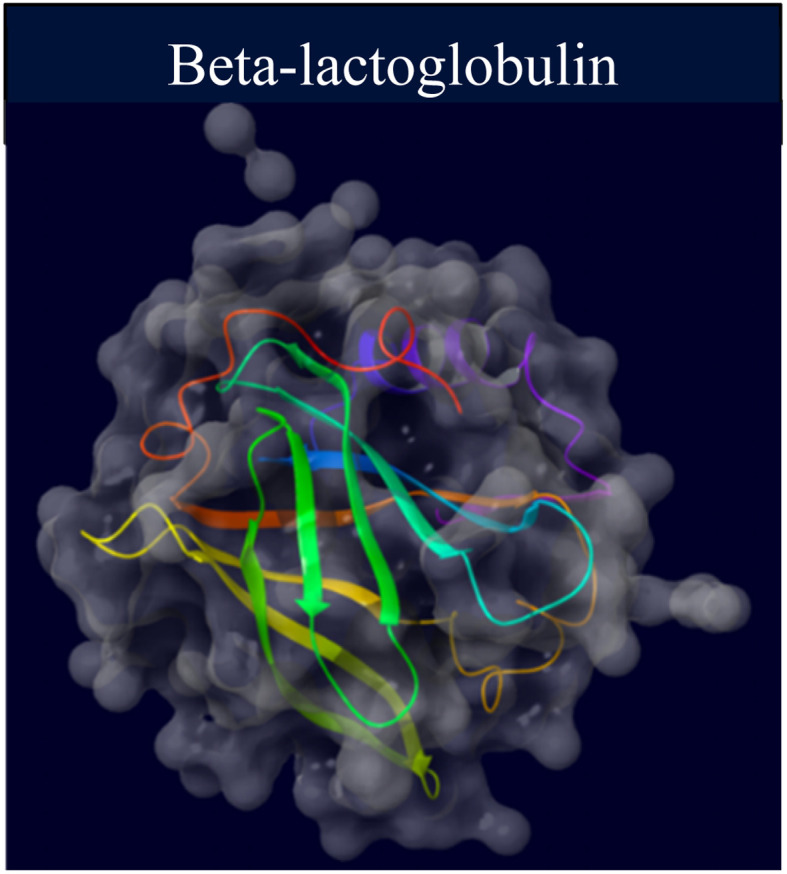
Schematic diagram of the crystal structure of BLG (PDB ID: 2Q2M and processed with the Maestro software).

BLG is a globular protein soluble in water, made up of 162 amino acid residues, present in the milk of most mammals. It possesses a β-sheet-rich secondary structure (50%) with nine antiparallel strands that form a hydrophobic ligand-binding pocket called the calyx, and one short and one long helix at the carboxyl end (Fig. 1). It plays a crucial role in human nutrition, serving as a protein source and as a carrier for retinol and fatty acids.^48^ Previous studies have availed of BLG as a model to elucidate the mechanism of protein folding.^49,50^ As mentioned, the native structure of BLG has been demonstrated to interact with various hydrophobic ligands and polycyclic compounds, and aromatic compounds, and its structure and unfolding transitions have been extensively analyzed through various physicochemical studies.^51^

As previously stated, this study investigates the interaction between gelatin-derived carbon quantum dots (gelatin-derived CQDs) and the beta-sheet-rich whey protein, examining their impacts on the structure and binding affinity to ligands. We also explore the influence of CQDs on BLG in folded, partially unfolded, and unfolded states, which will help provide an understanding of whether CQDs can restore the protein's native structure or exacerbate the unfolding processes. Furthermore, considering the potential of CQDs as biomedical tools, we will assess the safety of these CQDs on the viability of a neuroblastoma cell line, which is widely employed for underscoring the emergence and progression of neurodegenerative disorders.

## 2 Materials and methods

### 2.1. Chemicals

Commercially sourced chemicals included Tris–HCl (Millipore-Sigma, MO, USA), BLG, urea (Fisher Scientific, NJ, USA), gelatin (from porcine skin) (Sigma-Aldrich, MO, USA), cell culture media DMEM/F-12 (Dulbecco's Modified Eagle Medium/Nutrient Mixture F-12) (Millipore-Sigma, USA), Hoechst 33342 fluorescent stain (Invitrogen, Carlsbad, CA, USA), propidium iodide (PI) (Invitrogen, Eugene, OR, USA), fetal bovine serum (FBS) (Atlanta Biologicals, Atlanta, GA, USA), dialysis bags (3 kDa) (Fisher Scientific, Waltham, MA, USA), and microfilters (0.22 μm) (Fisher Scientific, Waltham, MA, USA). High-purity de-ionized water (18 MΩ cm^−1^ resistivity) from a Milli-Q water purification system (Millipore, Bedford, MA, USA) was used to prepare all solutions, including gelatin-CQDs and BLG.

### 2.2. Carbon quantum dots

#### 2.2.1. Synthesis of gelatin-sourced CQDs

To begin with, 0.6 g of gelatin powder was dissolved in 30 mL of Milli-Q water and heated on a hot plate at 40 °C with moderate stirring to aid in dissolution. The mixture was then transferred to a 50 mL hydrothermal bomb lined with Teflon and subjected to an oven treatment at 200 °C for 3 hours. After the treatment, the bomb was allowed to cool to ambient temperature. To remove larger nanoparticles, impurities, and aggregates, the yellowish solution was centrifuged at 15 000 rpm for 30 minutes, followed by filtration through 0.2 μm microfilters to obtain a light yellow aqueous solution. For long-term storage, the final product was freeze-dried.^44^

#### 2.2.2. Characterization of gelatin CQDs

Light absorbance readings were taken with a Genesys 10s UV-vis spectrophotometer (Thermo Scientific). Fluorescence measurements were obtained using a DM45 Olis spectrofluorimeter, which was maintained at 24 °C with a water bath. Dynamic light scattering (DLS) analyses were performed at ambient temperature using a Malvern Zetasizer Nano ZS90. Infrared (IR) data were acquired with a BRUKER spectrometer (Tensor 27, USA). Transmission electron microscopy (TEM) imaging of CQDs was carried out using high-resolution TEM (HR-TEM, JEOL 2010F, UNAM University).

### 2.3. Fluorescence measurement

To evaluate the impact of CQDs on the tertiary structure of proteins, fluorescence spectra were measured in the range of 300 to 400 nm in scan mode (bandwidth of 0.5 nm; excitation at 280 nm) on a DM45 Olis spectrofluorimeter (25 °C). Emission spectra were obtained by dissolving 1 mg per mL protein in a 100 mM phosphate buffer (pH 7.0). Native-state BLG was pre-treated (30 minutes prior to obtaining the spectra) with increasing concentrations of gelatin-derived CQDs (0, 0.1, 0.15, 0.2, 0.35, 0.5, 0.7, and 1 mg mL^−1^). For partially unfolded/unfolded state studies, the protein was treated with urea (1–6 M) before incubation with CQDs. Tryptophan fluorescence of CQDs–proteins at the maximum emission wavelength was adjusted for the inner filter effect using the formula below:*F*_1_ = *F*_0_ × e^((abs. 280 + abs. max emission)/2)^where *F*_1_ represents the adjusted fluorescence, *F*_0_ denotes the peak fluorescence measured, abs. 280 refers to the absorption at 280 nm, and abs. max emission indicates the absorbance at the peak emission wavelength.

The maximum fluorescence intensities of the protein alone and the protein bound to CQDs (excited at 280 nm) were recorded to monitor any shift or change in intensity in the maximum fluorescence wavelength.

### 2.4. Circular dichroism

To investigate the secondary structure of proteins subjected to varying concentrations of CQDs (0, 0.1, 0.2, 0.35, 0.5, 0.7, and 1 mg mL^−1^), circular dichroism (CD) spectra were obtained using a JASCO J-1500 spectrometer (USA) at 25 °C. The spectra were recorded with the following parameters: a bandwidth of 1 nm, a step size of 1 nm, a scan rate of 50 nm min^−1^, and a slit width of 0.02 mm. Far-UV measurements in the range of 190–260 nm were performed using a quartz cell with a 0.1 mm path length. Different concentrations of gelatin-sourced CQDs (0 to 1 mg mL^−1^) were mixed with 1 mg mL^−1^ (5 μM) protein in a 5 mM Tris–HCl buffer or 1.5 M urea for partially unfolded proteins. The protein solution was exposed to CQDs for 30 ± 5 minutes before spectral recording. The spectra were recorded and averaged through three scans of triplicate samples. Triplicate scans of the control buffer (5 mM Tris–HCl buffer) were also gathered, averaged, and subtracted from the sample spectra.

### 2.5. Fourier transform infrared (FTIR) spectroscopy

BLG (5 μM) was dissolved in 5 mM Tris–HCl. FTIR spectra were recorded in absorbance mode using a BRUKER Tensor 27 FTIR spectrometer. Subsequently, the protein was mixed with CQDs to prepare identical protein concentrations, albeit with different concentrations of CQDs. Data were collected 30 minutes after CQDs were added. The buffer solution (5 mM Tris–HCl) served as the blank (background) spectrum. The spectra of the protein alone and the protein–CQD mixtures were individually adjusted by subtracting them from the background spectrum. The resulting data comprised absorbance spectra within the 4000–500 cm^−1^ spectral wave number range. The amide I region spectra (1700–1600 cm^−1^) were analyzed with Gaussian deconvolution in the FTIR spectrum using OriginPro software.

### 2.6. Cell culture

Human neuroblastoma cells (SH-SY5Y; ATCC, Manassas, VA) were maintained in a cell culture medium composed of DMEM/F-12 (398225 SIGMA), enriched with 10% fetal bovine serum and 1% penicillin. The cells were cultured by maintaining them in a T75 flask and incubating at 37 °C with 5% carbon dioxide. The experiments involved seeding cells into a 24-well plate and allowing them to incubate until confluency was acquired. Upon reaching confluency, the cells were exposed to synthesize CQDs at concentrations ranging from 100 μg mL^−1^ to 10 mg mL^−1^ for 24 hours.^52–54^ In addition to the Ge-CQD treatment, groups including untreated (negative control), vehicle (H_2_O), and hydrogen peroxide (H_2_O_2_) (positive control) were also incubated in the same plate for 24 hours before undergoing microscopic analysis.

#### 2.6.1. Measuring cytotoxicity

Cytotoxicity of gelatin-derived CQDs was determined to obtain the CC_50_. The cytotoxic effect was measured by seeding 10 000 cells per well into 96 well plates. After the cells reached 90% confluency, different ranges of CQDs (100 μg mL^−1^ to 10 mg mL^−1^) were introduced to the wells. The treatments on the plate were categorized as follows: a control group for baseline comparison, H_2_O as the vehicle, and hydrogen peroxide as the insult. One hour prior to taking readings, each well was treated with a dye mixture of Propidium Iodide (PI) and Hoechst 33342, both at a final concentration of 1 μg mL^−1^. Images were captured in live-cell mode using a multi-well plate reader, specifically the BD Pathway 855 confocal automated microscope system (GE Healthcare) with a 10× objective lens, and managed with BD Pathway Analyzer 2000 Acquisition v4.0 software (GE Healthcare). For each well and fluorescence channel, four contiguous fields were captured in a (3 × 3) montage. Image acquisition and data analysis were conducted using BD Pathway Analyzer Workstation v3.7.2 software (GE Healthcare), which facilitated image segmentation to define regions of interest and calculate the cytotoxicity percentages of cell death for each well.

### 2.7. Statistical analysis

The data were expressed as mean ± standard deviation (SD) and analyzed with IBM SPSS Statistics 22 software. A one-way ANOVA with subsequent post-hoc testing was performed for all experiments. Statistical comparisons were made at significance thresholds of 5% (*P* < 0.05) and 1% (*P* < 0.01).

## 3 Results and discussion

### 3.1. Gelatin CQD characterization

**Fig. 2:**
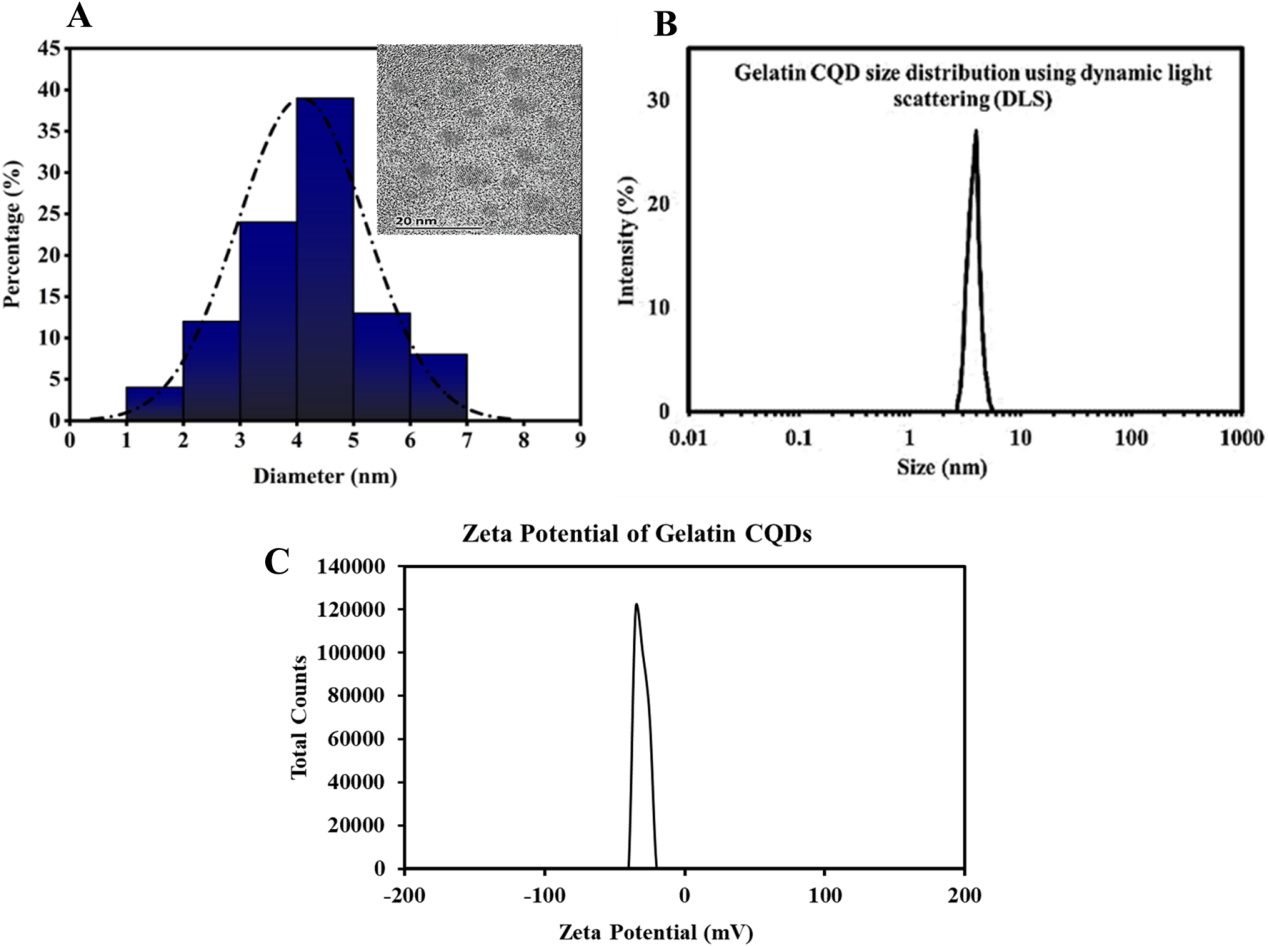
(A) Gaussian distribution of particles was derived from HR-TEM images, showing an average size of approximately 4 nm. The inset displays HR-TEM at a 20 nm scale. (B) The DLS profile of gelatin CQDs. (C) Zeta potential curve of the as-synthesized CQDs. The CQDs showed a negative surface charge of −32 mV.

The exclusive optical characteristics of CQDs, including absorption and emission peak wavelengths, bandwidths, and distinct electrical properties, such as the type of charge carriers (holes or electrons), derive from the chemical composition and extremely small size of these particles.^55^ In this study, we used gelatin as a precursor for CQDs, and detailed information about their size is presented in Fig. 2. The CQDs showed homogeneity in size, as observed by TEM and confirmed by DLS measurements. Based on the TEM results, the gelatin-derived CQDs had an average size of 4.12 nm, dispersed between 1.7 and 7.1 nm. The size distribution analysis showed a Gaussian distribution, with 38% of the particles clustered around 4 nm and over 85% of the CQDs ranging between 2 and 6 nm in size (Fig. 2A). This range aligns well with previously reported values for CQDs. DLS measurements (Fig. 2B) indicated an average size of ∼4.5 nm, with a range of dispersion from 2.8 to 5.5 nm. The zeta potential measurements indicated that the CQDs exhibit a potential of −32 mV (Fig. 2C), which helps prevent particle agglomeration and suggests a relatively high stability. The negative zeta potential is likely due to the presence of carboxylic acid functional groups on the surface of the CQDs.

**Fig. 3:**
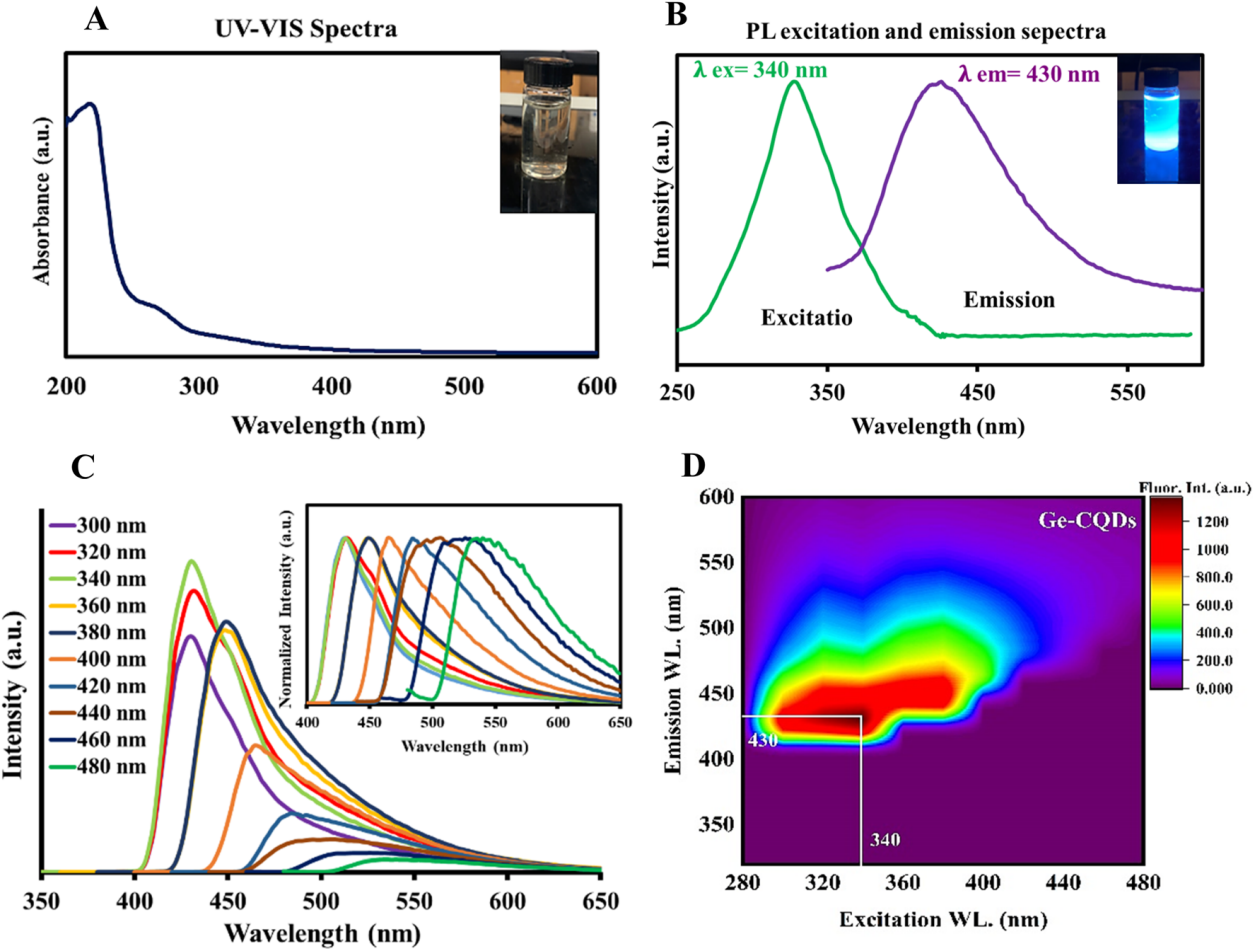
(A) UV-vis absorption spectrum and corresponding digital image, (B) the photoluminescence (PL) emission and excitation spectrum, and (C) PL spectra of the CQDs with different excitation wavelengths; inset: corresponding normalized PL emission. (D) Fluorescence contour map of gelatin CQDs.

To assess the optical properties of the gelatin-sourced CQDs, UV-vis absorption and fluorescence spectra were obtained. As illustrated in Fig. 3A, the absorption spectrum shows a broad peak in the range of 250–290 nm, which is characteristic of an aromatic system and the π–π* transition of the carbonyl group.^56^ The aqueous suspension of CQDs displayed a vivid blue fluorescence under UV light, visible to the naked eye (inset of Fig. 3A). A broad emission with *E*_max_ = 430 nm was observed in the fluorescence spectrum when excited at 340 nm (Fig. 3B). The spectral overlap between the intrinsic fluorescence emission of BLG and the excitation spectrum of CQDs suggests the possibility of Förster resonance energy transfer (FRET). However, the observed enhancement in CQD fluorescence in this study is more likely attributed to the direct binding interactions between BLG and CQDs, which may enhance the intrinsic fluorescence properties of CQDs rather than result from energy transfer. Although FRET could theoretically occur under such conditions, its efficiency depends on the precise spatial arrangement and distance between BLG and CQDs, which may not consistently fall within the Förster distance (1–10 nm). Additionally, no direct evidence of FRET, such as a measurable reduction in BLG fluorescence lifetime, was observed in this study. Thus, the fluorescence enhancement is attributed to binding-induced effects rather than energy transfer mechanisms. A suspension of CQDs in water showed a blue emission under UV light, readily visible to the naked eye (inset to Fig. 3B). The CQDs also demonstrated an excitation-dependent shift in emission. As the excitation wavelength was varied from 300 to 480 nm (Fig. 3C), the peak emission exhibited a gradual red-shift, along with a decrease in intensity. Fluorescence in CQDs primarily arises from surface-defect states caused by various surface-functional groups, leading to multicolor emissions and excitation-dependent properties.^57,58^ Defects, such as surface imperfections and heteroatom doping, greatly affect fluorescence properties by serving as exciton traps, which influence the energy gap and contribute to fluorescence associated with surface states. The data indicate the existence of defect centers and/or multiple fluorophores within the CQDs, a common observation in such nanomaterials.^59,60^ A contour plot illustrating fluorescence emission intensity was created by varying the excitation wavelength from 280 to 440 nm, with intensity depicted on a “*Z*-scale” (Fig. 3D). The data demonstrate that emission extends beyond 400 nm, even with excitation at 300 nm, suggesting the presence of highly conjugated systems with sp^2^ hybridized structures in the gelatin CQDs.^61^

In the XRD spectrum shown in Fig. S1A (ESI†), a weak and broad reflection peak was observed around 20°, suggesting an amorphous structure of synthesized CQDs. Analysis of the CQDs' IR spectrum showed strong agreement with the data reported in the literature (Fig. S1B†). The presence of carboxylic acid and other oxygen-rich functional groups is demonstrated by a broad O–H peak at 3400–3500 cm^−1^, a C=O stretching vibration band at 1700 cm^−1^, and a C–O stretching vibration band at 1100 cm^−1^.^62^ The absorption band at 3065 cm^−1^ is attributed to the N–H group,^63^ and the peak at 1450 cm^−1^ is attributed the vibrational and bending motions of N–H bonds, implying the presence of amino-functional groups. Finally, the absorption peaks at 1320, 1400, and 2920 cm^−1^ are linked to the stretching vibrations of C–C, C=C, and C–H groups, respectively, signifying the presence of alkyl and aryl groups.

### 3.2. Gelatin CQDs and tertiary configuration of β-lactoglobulin

Intrinsic fluorescence is a valuable tool for monitoring various protein-related events, including protein–ligand binding interactions, protein–protein interactions, and protein folding/unfolding processes.^64^ In this experiment, the intensity of tryptophan fluorescence and the wavelength (*λ*_max_) at which the intensity is maximum served as signals to monitor alterations in tertiary structure caused by varying concentrations of gelatin-sourced CQDs. Despite the crucial necessity to understand the kinetics of tertiary conformational changes in proteins or enzymes bound to nanoparticles, there is a limited body of research in this area, highlighting the significance of systematic exploration for developing nanoscale biotechnology.^65^

**Fig. 4:**
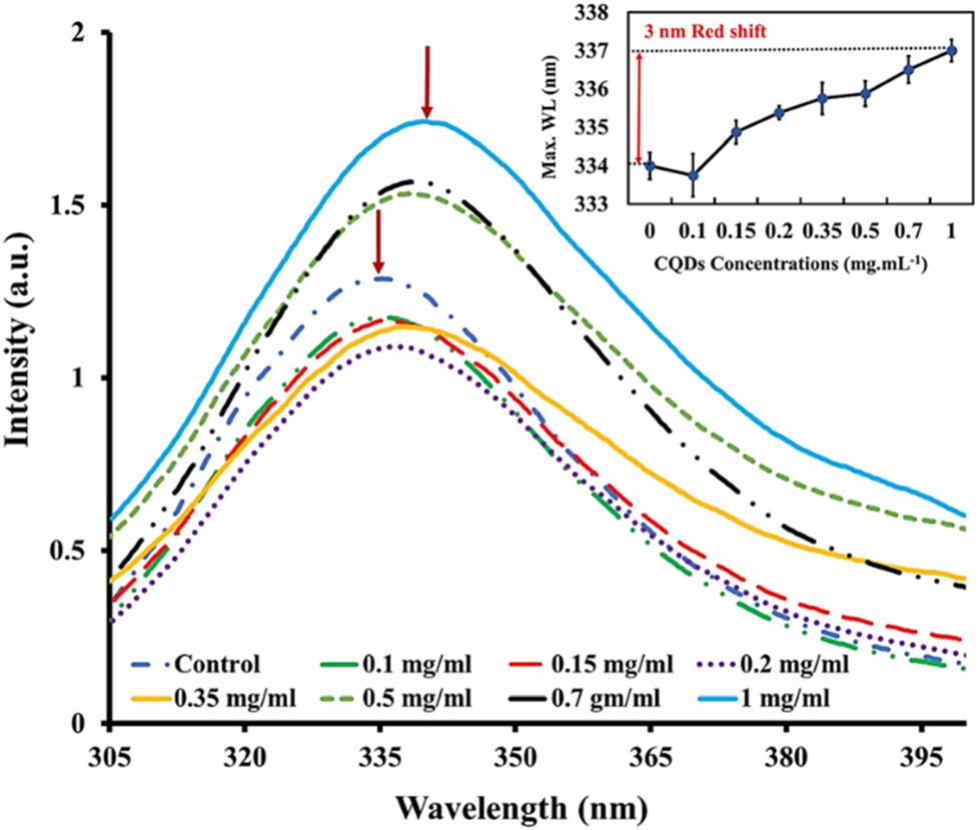
Steady-state fluorescence spectra for beta-lactoglobulin (*λ*_ex_ = 280 nm) when exposed to different concentrations of gelatin-sourced CQDs. Insets shows the emission maximum wavelength of beta-lactoglobulin in native state treated with different CQD concentrations.

To investigate the effect of CQDs on the tertiary structure of proteins, we analyzed the intrinsic fluorescence characteristics of BLG in response to varying CQD doses. The fluorescence emission spectrum of BLG treated with gelatin-derived CQDs (0.1–1 mg mL^−1^) is presented in Fig. 4. The emission intensity of BLG intrinsic fluorescence shows a dose-dependent increasing trend. Additionally, we observed a gradual red-shift in the maximum emission wavelength with increasing CQD doses. Specifically, a 3 nm red-shift in *λ*_max_ is noted, transitioning from 334 nm (in the absence of CQDs) to 337 nm (@1 mg per mL CQDs).

The interaction between CQDs and BLG is likely driven by the surface functional groups on the CQDs, which may form non-covalent interactions with specific amino acid residues in BLG, such as tryptophan, phenylalanine, and tyrosine. These interactions may involve hydrogen bonding, electrostatic forces, or π–π stacking, leading to alterations in the protein's conformation, as indicated by changes in its fluorescence.

BLG has two tryptophan residues, Trp-19 and Trp-61, situated in distinct environments within the protein structure.^66^ Trp-19 is located in a hydrophobic cavity within the protein, while Trp-61 presents at the molecule's surface and is located close to a disulfide bond. The primary source of intrinsic fluorescence in BLG is Trp-19, as it is located in a hydrophobic environment that shields it from solvent molecules. However, the fluorescence of Trp-19 can be affected by changes in the protein conformation or environment.^67^ The denaturation of BLG with urea leads to increased exposure of Trp-19 and Trp-61 to the aqueous environment, causing a red shift in the protein's intrinsic fluorescence emission. This shift is linked to the increased polarity of the environment around the Trp residues.^68^

**Fig. 5:**
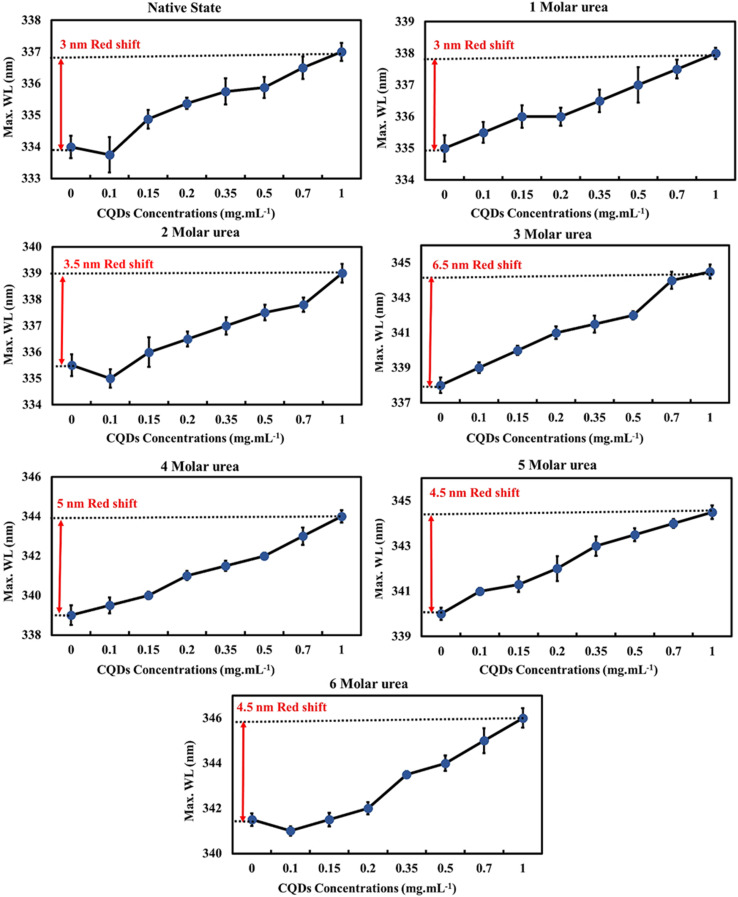
The emission maximum wavelength of beta-lactoglobulin at different urea concentrations (0–6 M) upon titration with gelatin CQDs.

In the presence of different concentrations of CQDs, the native protein showed a red-shift (3 nm) similar to partially unfolded protein (BLG in the presence of 1–2 M urea). However, at higher urea concentrations, the CQDs caused a more pronounced red-shift in *λ*_max_ (Fig. 5). This trend suggests that CQDs have a similar effect to denaturing agents on the globular protein, as evidenced by the enhancement in the fluorescence intensity of Trp residues (likely Trp19) by a reduction in its disulfide-associated quenching. Since the red-shift in *λ*_max_ suggests the unfolding of the tertiary structure and greater exposure of protein tryptophans, the CQDs appear to induce some degree of unfolding of BLG.

Next, we evaluated the impact of CQDs on different levels of unfolded protein (in the presence of 1–6 M urea) by examining alterations of BLG intrinsic fluorescence (Fig. S2†). Across all tested urea concentrations (0–6 M), increasing the CQD concentration increased the normalized fluorescence intensity of the protein, suggesting greater exposure of tryptophan, which is an indicator of protein unfolding. These data also suggest that CQDs induce some degree of protein unfolding even at higher urea concentrations. Furthermore, at higher urea concentrations (3–6 M), the *R*^2^ data suggest a stronger correlation between CQD concentration and intrinsic fluorescence of proteins.

### 3.3. Gelatin CQDs and the secondary structure of β-lactoglobulin

Circular dichroism (CD) spectroscopy is an optical and sensitive technique that utilizes chromophores' differential absorption of left- and right-circularly polarized light. It can be used to obtain information about the secondary structure, folding, and binding properties of proteins, providing structural insights into protein conformations. This technique has also been employed to determine structural changes in proteins' interaction with nanoparticles. Specifically, the average percentages of α-helices and β-sheets can be estimated from the CD spectrum in the far-UV region using empirical algorithms.^69–72^

**Fig. 6:**
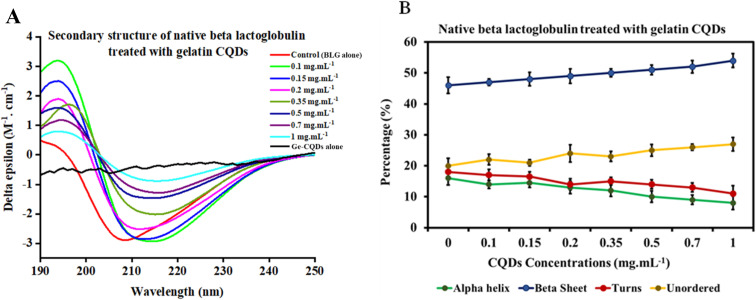
(A) CD spectra of beta-lactoglobulin alone, Ge-CQDs alone (1 mg mL^−1^), and BLG subjected to varying concentrations of gelatin CQDs. (B) The secondary structure and composition of native beta-lactoglobulin in response to different concentrations of gelatin CQDs.

The CD spectrum of native BLG was compared with that of BLG treated at different concentrations of gelatin-derived CQDs, as depicted in Fig. 6. An analysis carried out using the deconvolution program BestSel enabled the quantification of α-helix, β-sheet, turns, and unordered structures in BLG and BLG exposed to CQD treatment. The derived secondary structure shows a decrease in ellipticity at 208 nm with increasing concentrations of CQDs (0–1 mg mL^−1^; Fig. 6A). Detailed information, including RMSD and NRMSD, is provided in ESI Tables S1 and S2.† This data demonstrates how closely the CD measurements across the full wavelength range align with the theoretical CD spectra predicted from the calculated secondary structure composition. The deconvolution of CD spectra in the presence of increasing concentrations of gelatin-derived CQDs is presented in Fig. 6B. In response to increasing CQD doses, the helix content (green line) slightly decreases, while the parallel sheets show higher contents in 1 mg per mL CQDs. The turn and unordered contents remain relatively unchanged, and the differences are below the error of the measurement averages.

The *θ*_222_/*θ*_208_ ratio provides direct evidence of the conversion between single helices and coiled-coils (super helical confirmation). Ratios of 0.85 or below suggest single-stranded α-helices, whereas values of 1.0 or higher imply a fully folded coiled-coil structure.^73^ In the native state of BLG, a gradual rise in the *θ*_222_/*θ*_208_ ratio is observed with increasing CQD dosage (Fig. S3†). From these data, we can conclude that the interaction of gelatin-derived CQDs and BLG induces the transition of isolated helices into coiled-coil structures.

**Fig. 7:**
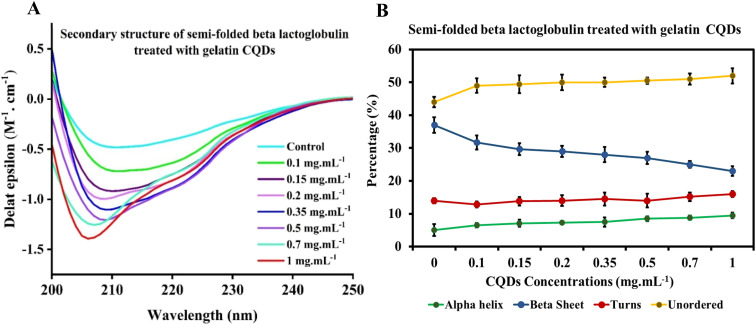
(A) CD spectra and (B) secondary structure composition of partially unfolded BLG exposed to increasing concentrations of gelatin-derived CQDs.

Moreover, we have examined the secondary structures of partially unfolded BLG before and after its interaction with gelatin-derived CQDs (Fig. 7). In the absence of CQDs, BLG treated with denaturant (1.5 M urea) exhibits a higher antiparallel sheet and lower α-helix content. The secondary structure components were altered after introducing 0.1–1 mg per mL CQDs into partially unfolded BLG, showing reduced antiparallel sheets, enhanced unordered structures, and a slight increase in helices (Fig. 7B).

The *θ*_222_/*θ*_208_ ratio in the partially unfolded state of BLG exhibited almost the same trend with increasing CQD doses (Fig. S4†). This ratio consistently remained below 1 for all treatments, indicating the presence of isolated helical structures in the partially unfolded state of the protein ± CQDs. This implies that the interaction between gelatin-derived CQDs and partially unfolded BLG did not lead to the bundling of helical structures, possibly due to insignificant changes in helical percentages before and after the interaction of partially unfolded BLG with CQDs.

It has been observed that some proteins retain their native-like structure once bound to the nanoparticle surface, while others undergo partial denaturation of their tertiary structure and may even experience disruption of their secondary structure.^74^ Adsorption of lysozyme or BLG onto silica nanoparticles resulted in a rapid alteration of both their secondary and tertiary structures.^61^ These interactions resulted in a decrease in α-helical content and an increase in β-pleated sheet content, indicating partial unfolding of the protein structure. Gold nanoparticles exhibit a strong affinity for critical blood proteins, including albumin, fibrinogen, α-globulin, histone, and insulin, often inducing conformational changes in these proteins.^75^ Similarly, a recent study investigated the interaction between vinegar-derived carbon dots (VCDs) and human plasma protein (HHB) using techniques such as FTIR, atomic force microscopy, and circular dichroism, revealing notable structural alterations in HHB upon binding with VCDs.^76^ A recent study revealed that CdSe/ZnS quantum dots can alter the secondary structure of insulin, leading to aggregation and fibrillation, with the extent of these effects influenced by the nanoparticles' size and surface charge.^35,77^

Zeta potential measurements of the Ge-CQDs alone showed that they possessed a negative surface charge under the experimental conditions, which likely contributes to the electrostatic and hydrophilic interactions with BLG. Although zeta potential measurements after CQD–BLG interactions were not performed, the structural and functional effects of these interactions were comprehensively analyzed using fluorescence spectroscopy and CD spectroscopy. This conformational change results from the protein's interaction with the nanoparticle surface, which can induce changes in the protein's shape and stability. Nanoparticles can cause disruption in protein structure through several factors, such as changes in the protein's hydration, exposure of hydrophobic residues, and steric hindrance of protein folding by the nanoparticle surface.^78^

### 3.4. Gelatin CQDs and functional groups of β-lactoglobulin

**Fig. 8:**
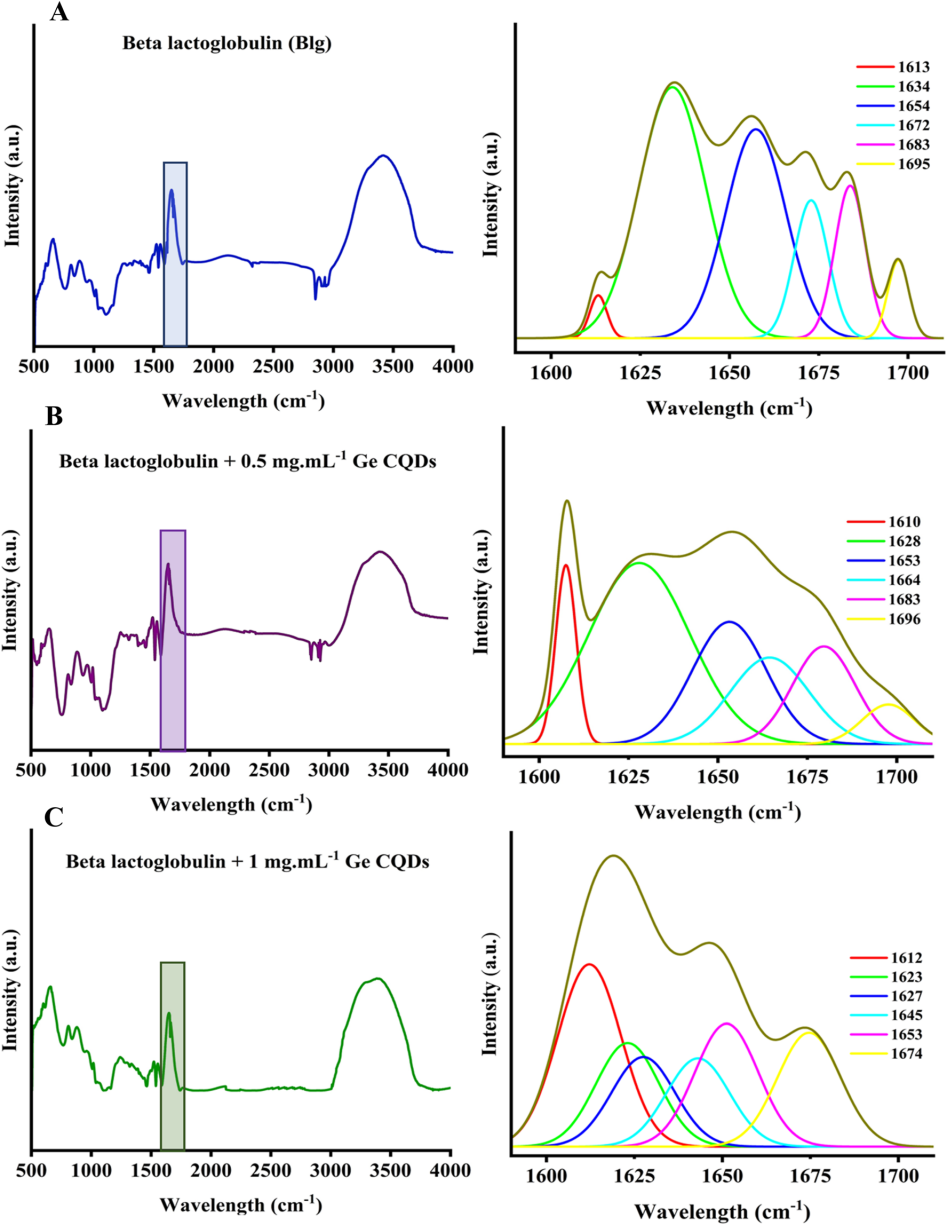
Deconvoluted IR spectrum of the amid I region in (A) IR spectra of BLG without gelatin CQDs, (B) BLG with 0.5 mg per mL gelatin CQDs, and (C) BLG with 1 mg per mL gelatin CQDs.

Direct information on the structure of BLG treated with gelatin-derived CQDs was obtained with FTIR spectroscopy, a confirmative technique to monitor the amide region of the compounds, and provides a deeper insight into the secondary structure components. Fig. 8 illustrates the complete IR spectra and deconvolution analysis of the amide I region for both native BLG and BLG treated with gelatin CQDs at concentrations of 0.5 and 1 mg mL^−1^, respectively. No significant alterations in this region were noted at concentrations below 0.5 mg mL^−1^ (data not presented here). The data obtained from BLG, both without and with 0.5 and 1 mg per mL gelatin-derived CQDs, aligned with the secondary structure data recorded by CD. According to the deconvolution analysis of the amide I region without gelatin CQDs, a Gaussian component (39% of the total peak area) centered at 1634 cm^−1^ is typically associated with β-sheet conformation. Gelatin-derived CQDs shifted this peak to 1623 cm^−1^ and 1628 cm^−1^ wavenumbers by 33% and 21% in 0.5 and 1 mg per mL gelatin-derived CQDs, respectively. The peak area centered at 1613 cm^−1^, ascribed to aggregated β-sheets, was 2% in the absence of CQDs (BLG alone), while in BLG treated with 0.5 and 1 mg mL^−1^ CQDs, this peak increased to 8% and 21%, respectively. The enhancement of the peak area at 1613 cm^−1^ indicates that CQDs induced some degree of aggregation in normal beta sheets of BLG. The dose-dependent increase of β-sheets in native BLG treated with CQDs is in agreement with these FTIR data, suggesting that higher concentrations of CQDs have greater effects on the aggregation of beta sheets. There was also an alteration in the peak area centered at 1654 cm^−1^, which is representative of the α-helix structure. This peak was reduced slightly from 28% to 24% in 0.5 mg mL^−1^ and 21% in 1 mg per mL CQDs. These findings indicate that the native structure of BLG experiences conformational changes when gelatin CQDs are present.

### 3.5. BLG–retinol binding rate and gelatin CQDs

Fluorescence quenching is a widely employed method to investigate the binding of ligands to proteins such as BLG.^79^ In this assay, the intrinsic fluorescence of a protein is used as a probe to monitor changes in protein conformation and energy transfer reactions that occur upon ligand binding. When a ligand binds to a protein, it can induce conformational changes that alter the fluorescence properties of the protein. In addition, energy transfer reactions can occur between the protein and the ligand, which can also affect the fluorescence properties of the protein.^80^ By monitoring the fluorescence of the protein upon ligand binding, quenching of fluorescence can provide information about the binding affinity, stoichiometry, and binding site of the ligand.^81^ Retinol–BLG interaction can cause quenching in fluorescence when the protein binds to retinol.^66^ In the case of BLG, its fluorescence emission is quenched when it binds to retinol because the protein molecules are in close proximity to the retinol molecule. The peak in the absorption of retinol occurs at approximately 325 nm when in the dilute solution in ethanol, and the BLG emission peak is around 330 nm. This proximity allows for energy transfer between the retinol and the protein, which leads to quenching of the fluorescence emission from the BLG.

**Fig. 9:**
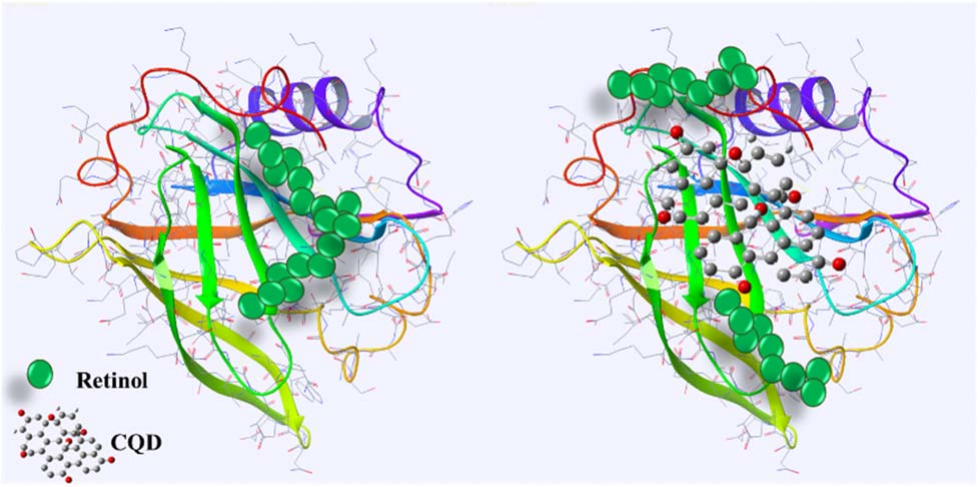
Schematic of the interaction of CQDs–BLG and disruption of retinol–BLG binding. Produced with the Maestro software.

**Fig. 10:**
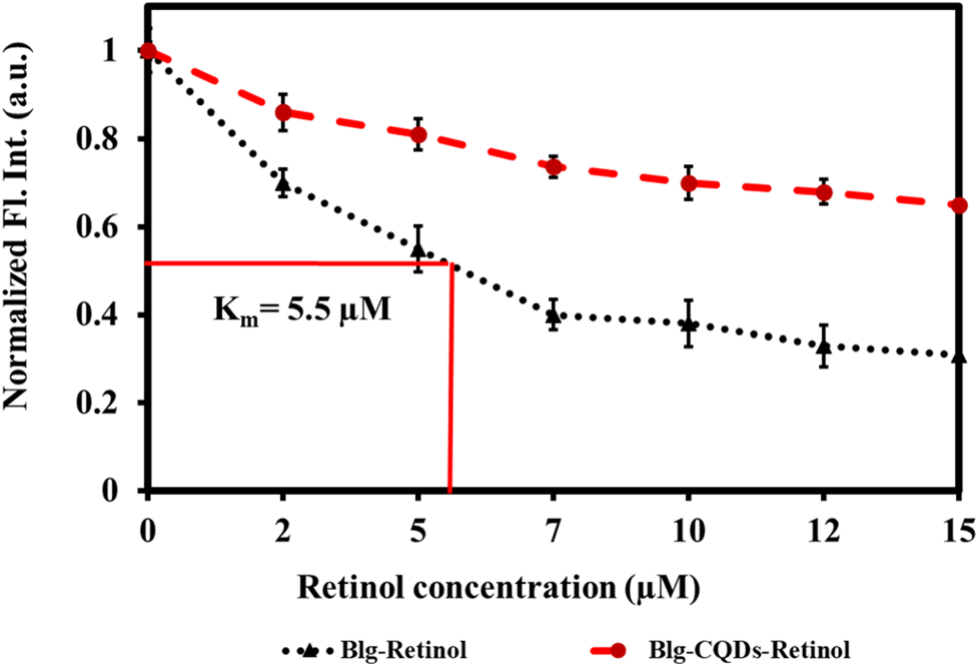
Relative fluorescence quenching of BLG by retinol as a response of BLG–retinol binding in the presence and absence of gelatin CQDs.

The results of our study showed that titration of BLG with increasing concentrations of retinol caused gradual fluorescence quenching, while pretreatment of protein with 1 mg per mL gelatin-derived CQDs caused reduced fluorescence quenching following titration with retinol (Fig. 10). This implies that CQDs disrupted the protein–retinol binding affinity. Retinol is the alcohol form of vitamin A and has been shown to bind to BLG, specifically on the protein's surface. Functional groups on the CQD surface might interact with the surface of BLG and occupy the binding sites for retinol (Fig. 9).

Studies have shown that the binding of carbon nanotubes (CNTs) onto proteins can have an impact on the proteins' native functions. For instance, research conducted by Park *et al.* showed that the size and shape of CNTs can effectively fit into the KcsA potassium channel, resulting in channel blockage and influencing its proposed function.^82^ Additionally, research by Karajanagi *et al.* investigated the effects of CNT binding on the activity of two enzymes, soybean peroxidase (SBP) and α-chymotrypsin (CT), after they adsorbed onto the single-walled carbon nanotubes. The results showed that both enzymes experienced a reduction in activity, with SBP retaining up to 30% of its native activity while CT retained only 1% of its native activity. Furthermore, SBP largely retained its structure, whereas CT experienced significant loss of its native form due to the exposure of hydrophobic surfaces, suggesting that the inhibitory activities of CNTs are closely related to those of the proteins.^83^ Another study by Yi *et al.* showed that CNTs modified with carboxylic groups interacted with ribonuclease A (RNase A), leading to a reduction in its enzymatic activity by altering its conformation.^84^ Furthermore, Zhang *et al.* developed surface molecular diversity to produce functionalized CNTs that can identify and attach to the catalytic site of α-chymotrypsin, resulting in the complete inhibition of its enzymatic activity.^85^ These findings suggest that the functionalization of CNTs can significantly impact their interaction with proteins, potentially affecting their activity and function.

While the interactions between surfaces and proteins are not well understood, it has been established that surface chemistry significantly influences protein adsorption.^86^ The effect of carbon nanomaterials on the structure of adsorbed proteins appears to vary based on the type of protein. Proteins interact dynamically with NPs over time, with their composition changing as the interaction progresses. This time-dependent process is explained by the Vroman effect, where initially adsorbed proteins, like fibrinogen, are replaced by higher-affinity proteins during longer exposure. The resulting protein layer consists of a “soft” layer of weakly bound proteins and a “hard” layer of strongly bound proteins.^87^ Factors such as the surface properties of NPs, the biological environment, exposure duration, and the physicochemical characteristics of the NPs all influence the composition of these layers.^88^ The results of our study demonstrated that the interaction of BLG with gelatin-derived CQDs has adverse effects on protein–ligand binding and protein function.

“The binding constant for the retinol : BLG complex was estimated at 5.5 μM (Fig. 10; black curve). When exposed to CQDs, there was an attrition in the binding strength of the complex as evidenced by the red trajectory. Within the limits of the experimental set-up, the binding constant of the complex in the presence of 1 mg per mL CQDs could not be determined. The CQD driven diminution in the binding of retinol to the protein can have a physiological impact because BLG is a transport protein for retinol and fatty acids for neonatal, infant and adult vision and brain development.”

### 3.6. Cytotoxicity of gelatin CQDs

**Fig. 11:**
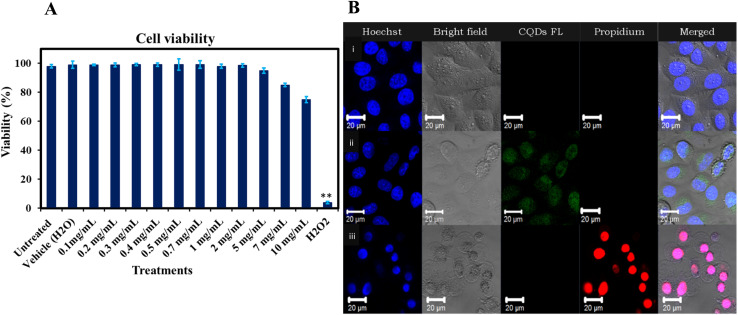
(A) Impact of gelatin-derived CQDs (0.1–10 mg mL^−1^) on the SH-SY5Y cell line viability.**The average difference is significant at the 0.01 level (*P* < 0.01). (B) Hoechst–PI dual staining images of SH-SY5Y cells: (i) untreated cells (negative control), (ii) cells treated with gelatin-derived CQDs, and (iii) cells treated with hydrogen peroxide (H_2_O_2_) (positive control).

The evaluation of gelatin CQDs' cytotoxicity involved the use of a neuroblastoma cell line (SH-SY5Y).^26^Fig. 11A presents the findings, showing that there are no significant alterations in cell viability with the addition of gelatin CQDs, even at a concentration of 5 mg mL^−1^, compared to the untreated and vehicle groups. Hydrogen peroxide (H_2_O_2_@10 μM) served as the positive control. These findings affirm the biocompatibility of gelatin CQDs at concentrations of up to 5 mg mL^−1^. In Fig. 11B, images of the control, CQD-exposed (5 mg mL^−1^), and H_2_O_2_ groups stained using PI and Hoechst dyes reveal live cells (blue color in merged image) and dead cells (red color in propidium iodide images). As demonstrated in Fig. 11B(ii), the cell viability in the CQD-treated group closely resembles that of the control group (Fig. 11B(i)), providing additional evidence of cell safety upon the introduction of CQDs. Surface modifications, such as pre-coating with proteins, can mitigate the cytotoxicity of nanomaterials. For instance, GO coated with bovine serum albumin (BSA) significantly reduced cytotoxic effects on A549 cells compared to uncoated GO.^89^ Furthermore, while GO exhibited concentration-dependent cytotoxicity at low fetal bovine serum (FBS) levels due to physical cell membrane damage, the presence of sufficient serum proteins altered these effects, reducing toxicity.^90^

### 3.7. Conclusion

Gelatin-derived CQDs were synthesized and characterized to evaluate their interaction with a model transporter protein, BLG, which predominantly contains beta-sheets in its secondary structure. The interaction of BLG with gelatin-derived CQDs resulted in a red-shift of the maximum emission wavelength and decreased tryptophan fluorescence quenching, indicative of protein unfolding. The secondary structure of native BLG exposed to gelatin-derived CQDs underwent significant changes. With increasing CQD doses, the helix content slightly decreased in native BLG, while the parallel sheets showed higher contents at 1 mg per mL CQDs. A gradually increasing *θ*_222_/*θ*_208_ ratio was observed as a function of CQD dose, indicating the transition of isolated helices into coiled coil structures.

Moreover, our study demonstrated that the interaction of BLG with gelatin-derived CQDs adversely affects protein–ligand binding, potentially causing disruption in protein function. Lastly, the observed low cytotoxicity of the CQDs aligns with prior findings. Additionally, consistent with other studies, the findings exhibited a concentration-dependent cytotoxic response of CQDs, highlighting the influence of CQD concentration on cytotoxicity against cells.

Our study investigated the intricate interactions between gelatin CQDs and structures of BLG, a model protein. Through the analysis of tertiary and secondary structural outputs and the examination of protein functional group signatures, we have advanced our molecular understanding of these interactions. The induced structural perturbations by gelatin-derived CQDs resulted in compromised functionality, yielding significant findings. This molecular insight adds to the broader understanding of carbon nanomaterial medicines, which have the potential to interact with a range of biomolecules beyond proteins, including DNA, RNA, carbohydrates, and lipids. The implications of these interactions on cellular function, particularly in terms of DNA replication, transcription, and repair, are crucial for assessing the safety and efficacy of nanomedicines.

While our findings contribute to the expanding knowledge of carbon nanomaterials for biomedical applications, more systematic and comprehensive research on their interactions is crucial. The intricate biological system of the human body necessitates a multifaceted approach, encompassing *in silico*, *in vitro*, and *in vivo* methods. Future studies on CNM–protein interactions at a molecular level could benefit from utilizing computational and experimental techniques, including molecular dynamics simulations, protein expression analyses, and X-ray examination of protein crystalline structures. Overall, our research findings suggest that a thorough understanding of how CQDs interact with biomolecules, particularly proteins, is essential for the development of safe nanomedicines.

## Data availability

All data will be made available upon request.

## Author contributions

SMA designed and conducted the experiments, analyzed the data, and wrote parts of the article. MN conceived the topic, designed some experiments, and wrote parts of the article.

## Conflicts of interest

There are no conflicts to declare.

## Supplementary Material

NA-OLF-D4NA00842A-s001
