# Supplementary material for: Surface interactions of gelatin-sourced carbon quantum dots with a model globular protein: insights into carbon-based nanomaterials and biological systems
Source: Nanoscale Adv. 2024 Dec 19;7(4):1104–17. doi: 10.1039/d4na00842a (PMC11694652; doi:10.1039/d4na00842a)
Supplement: NA-007-D4NA00842A-s001 [file NA-007-D4NA00842A-s001.pdf]

Supplementary Information (SI) for Nanoscale Advances.
This journal is © The Royal Society of Chemistry 2024

Surface Interactions of Gelatin-Sourced Carbon Quantum Dots with a Model Globular Protein: Insights into Carbon-Based Nanomaterials and Biological Systems

Shima Masoudi Asil[1], Mahesh Narayan[2]*

1. The Environmental Science & Engineering Program, The University of Texas at El Paso, El Paso, TX 79968, USA
2. The Department of Chemistry & Biochemistry, The University of Texas at El Paso, El Paso, TX 79968, USA

- *Corresponding email: mnarayan@utep.edu*

Electronic Supplementary Material Captions

Figures captions

Fig. S1. A) The XRD and B) FTIR spectra of gelatin and synthesized gelatin CQDs.

Fig. S2. The fluorescence emission maximum of beta-lactoglobulin in different urea concentrations upon titration with gelatin-CQDs, A) no urea, B) 1M urea, C) 2M urea, D) 3M urea, E) 4M urea, F) 5M urea, and G) 6M urea.

Fig. S3. The values of mean residue ellipticity at 208 and 222 nm for native beta-lactoglobulin treated with different concentrations of gelatin-CQDs, Inset shows Δε 222/208 ratios of native beta-lactoglobulin in different CQDs concentrations.

Figure S4. The values of mean residue ellipticity at 208 and 222 nm for semi-folded beta-lactoglobulin treated with different concentrations of gelatin CQDs. Inset shows Δε 222/208 ratios of semi-folded beta-lactoglobulin in different CQDs concentrations.

Tables Captions

Table S1. The secondary structure compositions of native beta-lactoglobulin treated with different concentrations of gelatin CQDs, estimated from their CD spectrum.

Table S2. The secondary structure compositions of semi-folded beta-lactoglobulin treated with different concentrations of gelatin CQDs, estimated from their CD spectrum.

Fig. S1

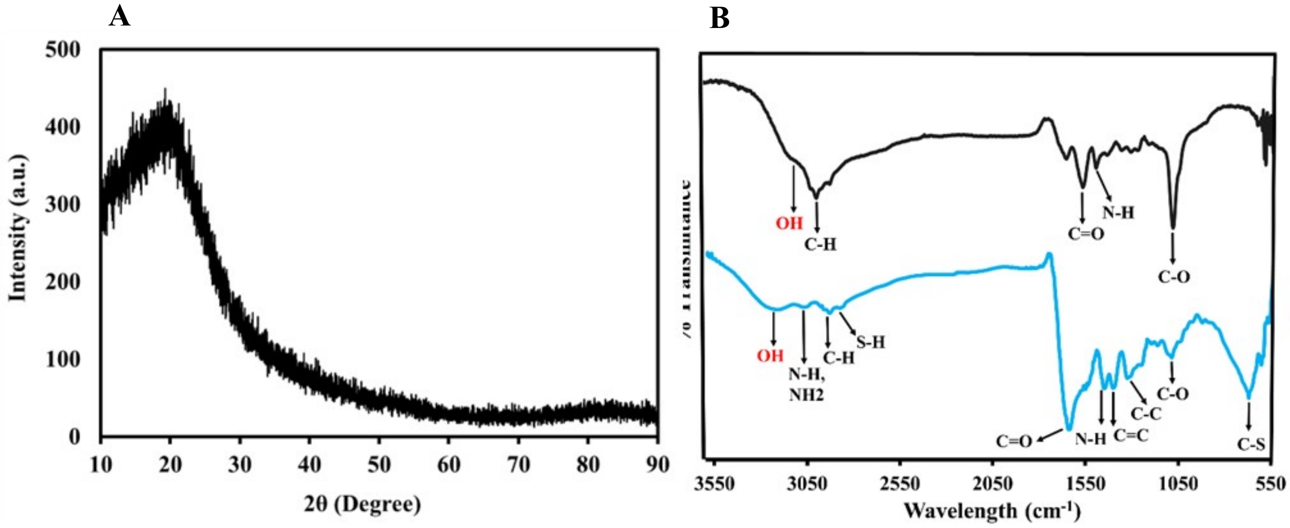
A
B
500
400
300
200
100
0
Intensity (a.u.)
10
20
30
40
50
60
70
80
90
2θ (Degree)
OH
C-H
C=O
N-H
C-O
OH
N-H,
NH2
C-H
S-H
C=O
N-H
C=C
C-C
C-O
C-S
3550
3050
2550
2050
1550
1050
550
Wavelength (cm-1)

Fig. S2.

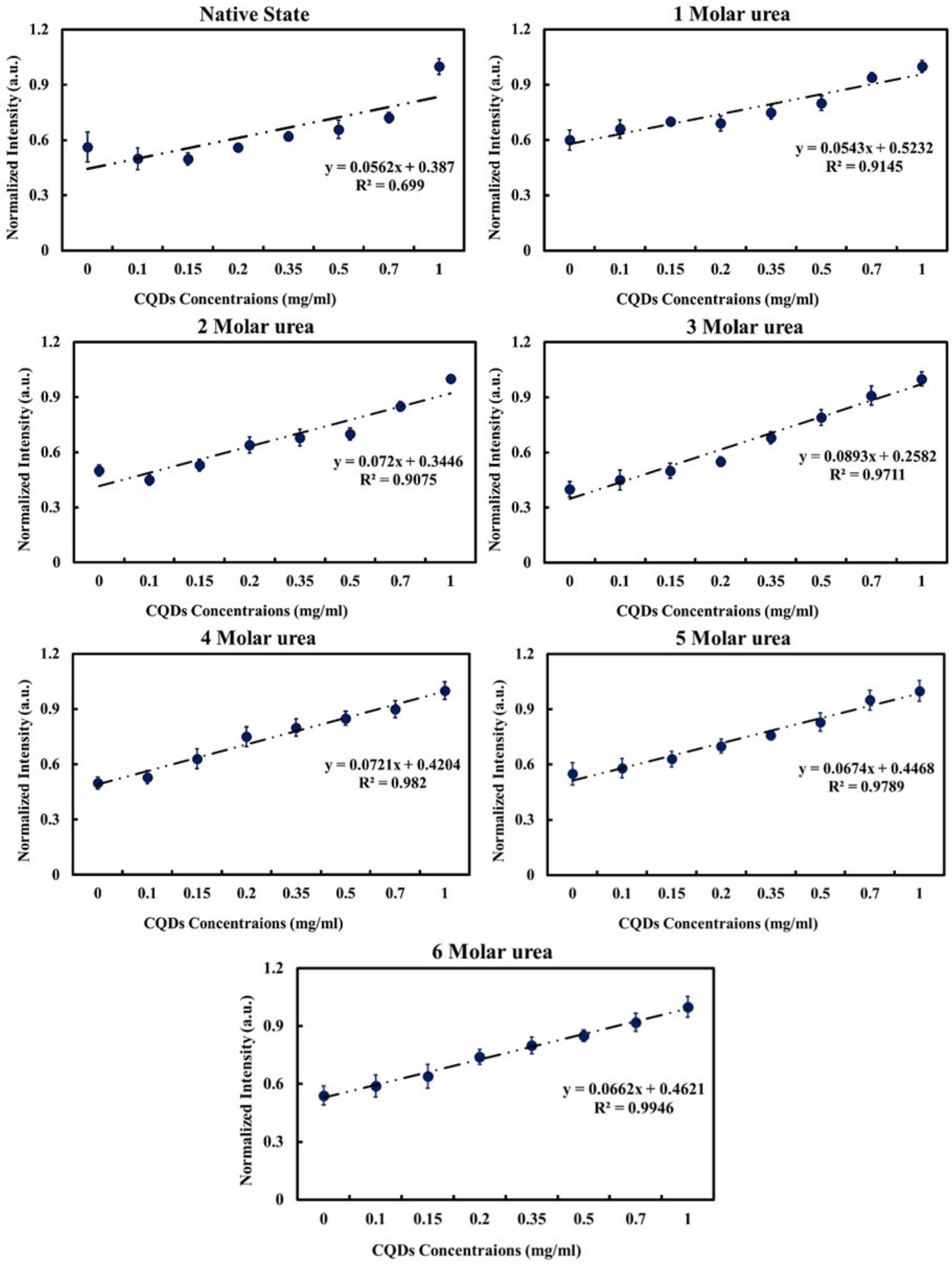
Native State
y = 0.0562x + 0.387
R² = 0.699
1 Molar urea
y = 0.0543x + 0.5232
R² = 0.9145
2 Molar urea
y = 0.072x + 0.3446
R² = 0.9075
3 Molar urea
y = 0.0893x + 0.2582
R² = 0.9711
4 Molar urea
y = 0.0721x + 0.4204
R² = 0.982
5 Molar urea
y = 0.0674x + 0.4468
R² = 0.9789
6 Molar urea
y = 0.0662x + 0.4621
R² = 0.9946
Normalized Intensity (a.u.)
CQDs Concentraions (mg/ml)
0
0.1
0.15
0.2
0.35
0.5
0.7
1
0.3
0.6
0.9
1.2

Fig. S3.

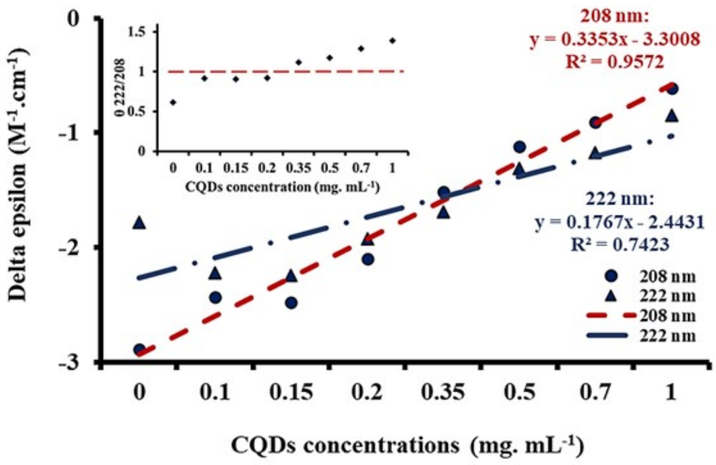

208 nm:
y = 0.3353x - 3.3008
R² = 0.9572
222 nm:
y = 0.1767x - 2.4431
R² = 0.7423
208 nm
222 nm
208 nm
222 nm
Delta epsilon (M-1.cm-1)
CQDs concentrations (mg. mL-1)
0 222/208
CQDs concentration (mg. mL-1)

Figure S4.

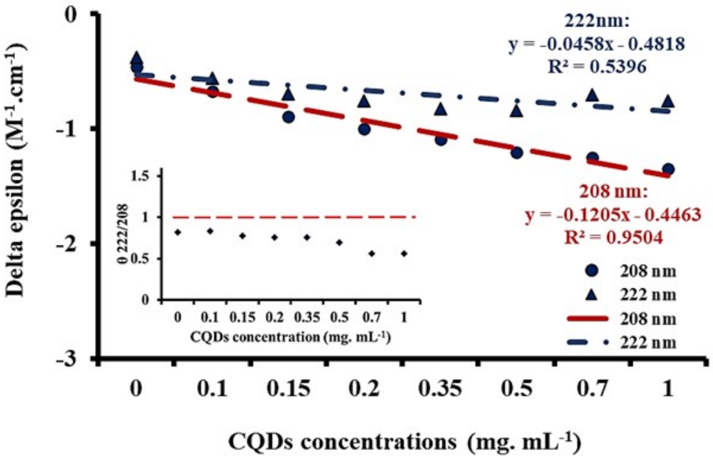
222nm:
y = -0.0458x - 0.4818
R² = 0.5396
208 nm:
y = -0.1205x - 0.4463
R² = 0.9504
Delta epsilon (M-1.cm-1)
CQDs concentrations (mg. mL-1)
208 nm
222 nm
θ 222/208
CQDs concentration (mg. mL-1)
0
-1
-2
-3
0.1
0.15
0.2
0.35
0.5
0.7
1
1.5
0.5

Table S1.

CQDs conc. (mg. ml^{-1}) / Items	0 (Control)	0.1	0.15	0.2	0.35	0.5	0.7	1	RMSD	NRMSD
Alpha Helix	16 ± 2.2	14 ± 1.3	14.5 ± 1.5	13 ± 2	12 ±1.9	10 ± 1.8	9± 1.5	8 ± 2	0.218	0.025
Parallel	33 ±1.3	34 ±1.5	33 ± 2	32 ± 2.2	31± 1.8	28± 2.4	28± 1.2	51 ± 1	0.453	0.042
Anti-parallel	13 ±1.6	15 ±1	15.5 ± 2.8	17 ±1.1	19± 0.5	23 ± 3.2	24±2	25 ± 1.7	0.300	0.011
Turns	18 ± 0.5	17 ± 1.8	16.5 ± 1.5	14 ± 1.7	15 ± 1.2	14 ± 1.4	13± 1.5	11± 2.6	0.255	0.013
Unordered	20 ±2.40	22 ±1.7	21 ± 1	24 ±2.8	23 ±1.6	25 ±1.9	26±1	27 ±2.1	0.446	0.028
Δε (222nm/ 208nm)	0.61	0.91	0.90	0.92	1.11	1.17	1.28	1.39	-	-

Table S2.

CQDs conc. (mg. ml-1) / Items	0 (Control)	0.1	0.15	0.2	0.35	0.5	0.7	1	RMSD	NRMSD
Alpha Helix	5 ± 1.8	6.5 ± 0.6	7 ± 1.1	7.3 ± 0.5	7.5 ±1.4	8.5 ± 0.7	8.8± 0.6	9.5±0.85	0.332	0.024
Parallel	11 ±1.2	10.7 ±0.5	10.7 ± 0.7	10 ± 1.5	11± 0.8	11± 1	12± 0.9	11 ± 0.76	0.405	0.039
Anti-parallel	26 ±2.4	21 ±2.1	19 ± 1.8	19 ±1.7	17 ± 2.3	16 ± 1.9	13± 1	12 ± 1.5	0.580	0.049
Turns	14 ±0.8	12.8 ±0.9	13.8 ± 1.3	14 ±1.6	14.5 ±2	14 ±2.1	15.2±1.3	16±0.9	0.415	0.040
Unordered	44 ±1.6	49 ±2.2	49.4 ± 2.7	50 ±2.4	50 ±1.4	50.5 ±1	51± 1.7	52 ±2.3	0.523	0.048
Δε (222nm/ 208nm)	0.82	0.83	0.77	0.75	0.76	0.69	0.56	0.56	-	-
